# Dataset for anomaly detection in a production wireless mesh community network

**DOI:** 10.1016/j.dib.2023.109342

**Published:** 2023-06-25

**Authors:** Llorenç Cerdà-Alabern, Gabriel Iuhasz

**Affiliations:** a Departament d’Arquitectura de Computadors (DAC), Universitat Politécnica de Catalunya - BarcelonaTech (UPC), Campus Nord, Edif. D6, C. Jordi Girona, 1-3, Barcelona 08034, Spain; b West University of Timisoara, Blvd. Vasile Parvan, Nr. 4, Timisoara, 300223, Romania

**Keywords:** Fault detection, Machine learning, Wireless network dataset, Wireless community networks

## Abstract

Wireless community networks, WCN, have proliferated around the world. Cheap off-the-shelf WiFi devices have enabled this new network paradigm where users build their own network infrastructure in a do-it-yourself alternative to traditional network operators. The fact that users are responsible for the administration of their own nodes makes the network very dynamic. There are frequent reboots of the networking devices, and users that join and leave the network. In addition, the unplanned deployment of the network makes it very heterogeneous, with both high and low capacity links. Therefore, anomaly detection in such dynamic scenario is challenging. In this paper we provide a dataset gathered from a production WCN. The data was obtained from a central server that collects data from the mesh nodes that build the network. In total, 63 different nodes were encountered during the data collection. The WCN is used daily to access the Internet from 17 subscribers of the local ISP available on the mesh. We have produced a dataset gathering a large set of features related not only to traffic, but other parameters such as CPU and memory. Furthermore, we provide the network topology of each sample in terms of the adjacency matrix, routing table and routing metrics. In the data we provide there is a known unprovoked gateway failure. Therefore, the dataset can be used to investigate the performance of unsupervised machine learning algorithms for fault detection in WCN. To our knowledge, this is the first dataset that allows fault detection to be investigated from a production WCN.

**Specifications Table**

**Table:** 

Subject	Computer Networks and Communications
Specific subject area	Anomaly detection using embedded router data from the wireless mesh nodes of a production community wireless network with 63 nodes.
Type of data	We provide two files with real-time data gathered every 5 min from every node in the mesh. The first one consists of node state information as CPU, load, processes, memory and traffic over each network interface. The network topology of each sample is provided in the second file in terms of the adjacency matrix, routing table and routing metrics.
How the data were acquired	To collect the data, a permanent ssh connection was established from a central monitoring server to each WCN node, which was used to execute standard system commands. The dump of the commands was then parsed to filter out the desired data.
Data format	Raw
	Parsed
Description of data collection	The data is obtained reading the linux kernel variables available through the /proc file-system. For instance, /proc/net/dev to read counters with the number of bytes and packets transmitted and received over each interface; /proc/stat where there is information about kernel activity; /proc/meminfo for memory usage, etc. Network topology is gathered using the information provided by the routing network deamon.
Data source location	Departament d’Arquitectura de Computadors (DAC), Universitat Politécnica de Catalunya - BarcelonaTech (UPC), Spain
Data accessibility	Repository name: Dataset for Anomaly Detection in a Production Wireless Mesh Community Network Data identification number: DOI 10.7910/DVN/NKTFZM Direct URL to data: https://doi.org/10.7910/DVN/NKTFZM
Related research article	Llorenç Cerdà-Alabern, Gabriel Iuhasz, and Gabriele Gemmi. Anomaly detection for fault detection in wireless community networks using machine learning. Computer Communications, 202 (2023) 191–203. https://doi.org/10.1016/j.comcom.2023.02.019

**Value of the Data**

• Datasets used in computer network research have often been synthetically manipulated, which has been criticised by some authors. A relevant example is the KDD dataset used in many intrusion detection research papers, where synthetic background and attack data are added [1]. In contrast, the dataset explained in this paper consists of an unmanipulated set of features collected from a production WCN.
• The dataset is useful for researchers interested in WCN, and fault detection analysis using real data.
• The dataset can be used to test unsupervised ML algorithms using a rich and heterogeneous set of features. Since we provide the network topology of each sample, the dataset could also be used to test graph neural network methods. Similarly, time-series forecasting and missing data imputation methods can also be tested/developed using this dataset.

## 1 Objective

Anomaly detection has received an increasing attention in the computer networking community. A main reason of this interest has been the recent advances of Machine Learning techniques. However, most work in the literature has focused on security issues as intrusion detection, denial of service attacks or malware detection. Limited attention has been given to the use of ML for fault detection, the main reason being the lack of datasets. The dataset presented in this paper attempts to fill this gap by providing measurements of a production WCN, collecting traffic and non-traffic features, e.g. CPU and memory. A live monitoring page of this WCN is available in GuifiSants [2]. An unprovoked gateway failure occurs during the data measurements, providing a known fault that can be used to estimate the accuracy of the ML models under study. Indeed, a research of the performance of 4 unsupervised ML approaches based on different principles using this dataset has been carried out in Cerdà-Alabern et al. [3]. In addition, the dataset presented in this paper extends the dataset used in Cerdà-Alabern et al. [3] by adding network topology information, including routing protocol metrics, routing tables and adjacency matrices. We believe that this data can be useful to study a wider set of ML methods, e.g. graph neural network methods.

Additionally, as the dataset is multi-variate, comprised of multiple time-series, ML methods for analysing time-series can also be easily applied. These methods include but are not limited to; cycle and pattern detection, forecasting using regression and missing data imputation. We should note that for these methods to be applied some additional pre-processing steps could be necessary such as windowing.

## 2 Data Description

**Fig. 1:**
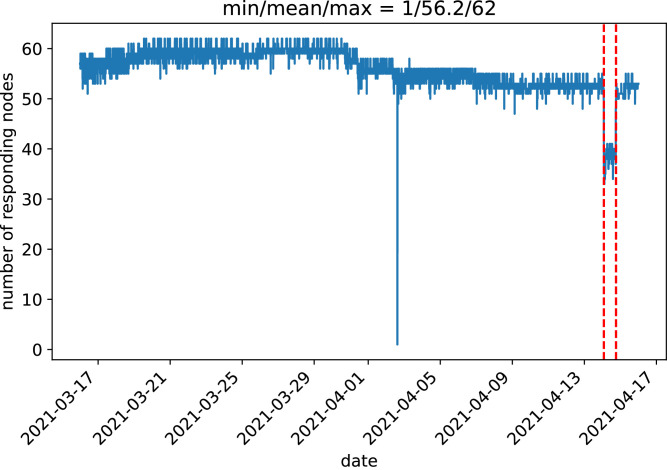
Graph of the number of responding nodes in each sample. Dashed lines show the gateway failure interval.

The dataset consists of two files, one of scalars with the general state of the nodes, and another of matrices with the network topology. In total the dataset contains 7931 samples collected in the interval from 2021-03-16 to 2021-04-15 (31 days). For each sample the first file provides 2387 features, 38 per node, the second file provides 3 matrices. The contents of the two files are described in detail below. On April 14, 2021, one of the two gateways available in the WCN (with nodeid 24) failed and was replaced. Due to the failure, 122 samples from this node were missing between 02:00 and 17:50 (both included) of April the 14th. Note that there may be nodes that join or leave the WCN, and nodes that may not appear in a capture, because they have been rebooted or temporarily shut down by a user. However, gateways are critical devices in the WCN, and their failure should be detected as an anomaly. The server gathering the data has a timeout period to get a response from a node. Therefore, even if a node is alive, its data may not be collected if it takes too long to respond. Fig. 1 shows the number of responding nodes in each sample. The figure also shows the gateway failure interval. Some noise can be observed due to several nodes being alive but not responding in some samples. Fig. 1 shows that there is one sample in which only one node responds. Clearly, this is an outlier where the server failed to connect to the other nodes of the mesh. During the gateway outage some nodes were disconnected from the mesh, as can be seen in Fig. 1. In total, 63 different nodes were encountered during the data collection, of which 56.2 responded, on average, to the server collecting the samples.

The first file is a compressed CSV text file. The first line of the file is a header describing the features. The first column is a GMT timestamp of the sample in the format as 2021-03-16 00:00:00. The rest of the columns provide the comma-separated values of the features collected from each node in the corresponding capture. A suffix with the node-id is added to each feature. For instance, the feature having the number of processes of node with node-id 24 is named as processes-24. Whenever possible, for every node in the WCN the dataset provides the features listed in Table 2. If a node does not have a feature, e.g. a node may not have WiFi interfaces, then the corresponding column is not present for this node. Features in Table 2 are ordered as in the dataset file. Whenever a feature was not recorded, it is left empty in the dataset. For instance, the values of the features of a node which was not reachable during a capture are left empty in the corresponding sample. Features are of two types: (i) absolute values, for instance, the CPU 1-min load average, and (ii) counters that are monotonically increased, for instance the number of transmitted packets. We have converted counter-type kernel variables to rates, by dividing the difference between two consecutive samples, over the difference of the corresponding timestamps in seconds. We have removed negative rate samples that occur when a node is rebooted, or when a counter reaches its maximum value and it is restarted. Counters type features in Table 2 have the suffix rate. The feature values listed in Table 2 were obtained from parsing the output of the commands indicated by the column source, as shown in Table 3.

**Table 2:** Features in the dataset file recorded whenever possible for each node.

Feature	Source	Description
processes	PROC	number of processes
loadavg.m1	UPT	1 min load average
softirq.rate	STAT	servicing softirqs
iowait.rate	STAT	waiting for I/O to complete
intr.rate	STAT	interruptions
system.rate	STAT	processes executing in kernel mode
idle.rate	STAT	twiddling thumbs
user.rate	STAT	normal processes executing in user mode
irq.rate	STAT	servicing interrupts
ctxt.rate	STAT	total number of context switches across all CPUs
nice.rate	STAT	niced processes executing in user mode
nr_slab_unreclaimable	MEM	Slab that can’t be reclaimed under memory pressure
nr_anon_pages	MEM	anonymous memory pages
swap_cache	MEM	Memory swapped back, but still in the swapfile
page_tables	MEM	Memory used to map between virtual and physical addresses
swap	MEM	Memory swapped out
eth.txe.rate	DEV	tx errors over all ethernet interfaces
eth.rxe.rate	DEV	rx errors over all ethernet interfaces
eth.txb.rate	DEV	tx bytes over all ethernet interfaces
eth.rxb.rate	DEV	rx bytes over all ethernet interfaces
eth.txp.rate	DEV	tx packets over all ethernet interfaces
eth.rxp.rate	DEV	rx packets over all ethernet interfaces
wifi.txe.rate	DEV	tx errors over all wireless interfaces
wifi.rxe.rate	DEV	rx errors over all wireless interfaces
wifi.txb.rate	DEV	tx bytes over all wireless interfaces
wifi.rxb.rate	DEV	rx bytes over all wireless interfaces
wifi.txp.rate	DEV	tx packets over all wireless interfaces
wifi.rxp.rate	DEV	rx packets over all wireless interfaces
txb.rate	DEV	tx bytes over all ethernet and wifi interfaces
txp.rate	DEV	tx packets over all ethernet and wifi interfaces
rxb.rate	DEV	rx bytes over all ethernet and wifi interfaces
rxp.rate	DEV	rx packets over all ethernet and wifi interfaces
sum.xb.rate	DEV	tx+rx bytes over all ethernet and wifi interfaces
sum.xp.rate	DEV	tx+rx packets over all ethernet and wifi interfaces
diff.xb.rate	DEV	tx-rx bytes over all ethernet and wifi interfaces
diff.xp.rate	DEV	tx-rx packets over all ethernet and wifi interfaces

**Table 3:** Commands run to get the feature values.

Source	Command
PROC	echo /proc/[0–9]*|wc -w
UPT	cat /proc/uptime
STAT	cat /proc/stat
MEM	cat /proc/meminfo
DEV	cat /proc/net/dev

The second file is a compressed python pickle file containing a dictionary with the 3 lists of matrices described below. Recall that pickle is a popular Python object storage format. When loaded, the data is contained in a standard Python dictionary. The matrices are Python numpy arrays. In each of the matrices the row is the origin, and the column is the destination. Each element in the three lists corresponds to the value of the matrices gathered in the corresponding sample provided in the first file.

• adj: Adjacency matrices. It is a matrix of booleans where the element is True if the destination is neighbor of the origin and False otherwise. Elements in the diagonal are set to False. Matrices might not be symmetric, because weak links might be unidirectional. If a node does not show up in the capture, it has no neighbors, and it will be a row with all elements False.
• rt: Routing tables. For each origin in the table there is the node-id of the next node to reach the destination. In the diagonal, and in missing nodes (not responding) there is ‘−1’. Thus, there is a row of ‘−1’ for each missing node in a sample.
• metric: BMX6 routing protocol metrics. The metric measured by BMX6 is a kind of “bandwidth” to reach every other node in the mesh. So, the higher is the metric, the better is the connection. In the diagonal (self connection) there is the maximum metric (128,000,000,000). As in the rt matrices, there is ‘−1’ if a node is missing.

## 3 Experimental Design, Materials and Methods

The dataset presented in this paper has been gathered from a production WCN deployed in the quarter of Sants, in Barcelona, Spain. The WCN is called GuifiSants [4], and it is part of a larger community network called Guifi.net [5]. To join GufiSants, users first register in Guifi.net [5] site and create nodes in the zone assigned to Guifisants, obtaining unique private IPv4 addresses within Guifi.net. Users are then responsible for installing the infrastructure on their rooftops to connect to the network. Normally, they are helped by volunteers that have technical background and experience with such deployments. The main use of GuifiSants is Internet access, which is also provided by the users themselves in a legally declared local ISP association and member of RIPE, called eXo [6], [7].

Guifisants was started in 2009 and in 2012 nodes from *Universitat Politècnica de Catalunya* (UPC) joined the network supported by the EU CONFINE project [8]. Since Guifisants was started the number of nodes has been rather variable. There are users who joined the network and eventually leave it because they changed their residence, or because their connection was not good enough, and switched to optical fiber. Another reason is the incorporation of research nodes in the UPC premises, which only functioned during experimentation periods. A live monitoring page of Guifisants is available in GuifiSants [2], and an experimental analysis is available in Cerdà-Alabern et al. [9].

The hardware used in Guifisants are economic outdoor WiFi devices with sector antennas, and parabolic for point-to-point long shots. Most sector antennas are flashed with a linux openwrt distribution [10], using the BMX6 mesh routing protocol [11]. Normally parabolic antennas used in point-to-point links are left with the manufacturer’s firmware, which offer higher performance. These are connected to other openwrt devices running the routing protocol, and are seen as simple Ethernet links. Data is gathered only from openwrt nodes running the routing protocol.

To collect the data, a permanent ssh connection was established from a central server to each monitored node. Samples were taken every 5 min executing standard system commands. The dump of the commands was then parsed to filter out the desired data and build the dataset files (the CSV text file with the features listed in Table 3, and the matrices stored in the pickle file). Using a ssh connection for data gathering has the advantage that no changes or additional software need to be installed in the nodes. This is an important condition since the users are the owner of their nodes. Therefore, only the users’ permission is needed to install a public key to access the node with ssh for monitoring purposes.

## Ethics Statements

Guifisats users are aware and consent that UPC participates in the WCN for research purposes, collecting data that does not involve any personal information. Thus, we had permission to use GuifiSants data.

## CRediT authorship contribution statement

**Llorenç Cerdà-Alabern:** Conceptualization, Data curation, Investigation, Software, Writing – original draft. **Gabriel Iuhasz:** Conceptualization, Investigation, Software, Writing – original draft.

## Declaration of Competing Interest

The authors declare that they have no known competing financial interests or personal relationships that could have appeared to influence the work reported in this paper.

## Data Availability

Dataset for Anomaly Detection in a Production Wireless Mesh Community Network (Original data) (Dataverse).
